# In vitro renal artery stenting using a steerable guide wire navigated by a mobile electromagnetic field

**DOI:** 10.1016/j.jvscit.2025.101842

**Published:** 2025-05-16

**Authors:** Christian Zielasek, Jonas Lussi, Silvia Viviani, Vladimir Makaloski, Drosos Kotelis, Silvan Jungi

**Affiliations:** a Department of Vascular Surgery, Inselspital, Bern University Hospital, University of Bern, Bern, Switzerland; b ETH Zurich, Multi-Scale Robotics Lab, Zurich, Switzerland

**Keywords:** Aortic aneurysm, Aortic surgery, Basic surgical research, Endovascular surgery, Endovascular therapy, Magnetic steering, Radiation protection, Renal artery stenting

## Abstract

**Objective:**

Endovascular procedures require precise guide wire control that often requires auxiliary devices. Therefore, catheterization can be time- and resource-intensive. A novel technology involves using a mobile external electromagnetic field to manipulate guide wires remotely, eliminating the need for additional devices. This experimental study evaluates the feasibility of this technology in a representative three-dimensional model mimicking the human vasculature, comparing it with conventional means.

**Methods:**

We assessed the efficacy of a magnetically steerable guide wire (MGW) vs a conventional guide wire (CGW) in catheterizing the renal artery in a three-dimensional silicone model of the abdominal aorta.

**Results:**

A total of 20 procedures were performed, with 10 utilizing an MGW for renal artery catheterization and 10 using a CGW. Technical success was 100% for all procedures. The median procedure time was significantly shorter for MGW than for CGW (105 vs 181 seconds; *P* < .001). We observed a trend towards lower median fluoroscopic time (65 vs 101 seconds; *P* = .06) and lower median fluoroscopic dose (6.84 vs 10.98 mGycm^2^; *P* = .063) for MGW compared with CGW.

**Conclusions:**

Precise control of a steerable guide wire using a mobile external electromagnetic field is technically feasible in a three-dimensional silicone model of the human aorta. It facilitates *in vitro* renal artery catheterization and stenting and reduces both procedure and fluoroscopy time. This novel technology could further improve numerous endovascular procedures.

In endovascular procedures, precise control of a guide wire is essential to reach a target vessel or deliver a device to its site of action. It is particularly challenging to navigate through complex curvatures or steep angles of a blood vessel as a variety of auxiliary devices must be used sequentially. Despite their use, it can be extremely time-consuming to reach a desired target, thereby generating a significant amount of radiation exposure for patient and operator.^1^ In some cases, it is impossible to successfully catheterize a target vessel from the original access site. This leads to a necessity for special techniques that are subject to significant morbidity, such as upper extremity arterial access to reach reno-visceral arteries, or even to treatment failure because catheterization of the target vessel cannot be achieved.^2^^,^^3^ This is especially true for complex endovascular aortic procedures, where difficult anatomy with steep arterial angles may necessitate open surgical or highly material—and therefore cost-intensive bailout strategies.^4^^,^^5^

Employment of robotic catheterization systems that allow for precise three-dimensional control of a guide wire can partially overcome these difficulties and enable a degree of steerability currently unattainable with conventional measures. Several systems have been developed.^6^, ^7^, ^8^ However, they are not widely used in clinical practice, primarily due to high costs and challenges with integration caused by the bulkiness and weight of these systems.^9^

The system currently under investigation does not employ passive permanent magnets but rather an active electromagnetic field generator, thereby significantly increasing environmental safety. It features a proprietary cooling technology, which allows for the creation of smaller yet more powerful magnets within a single unit, eliminating the need for two opposing, rotating machines with the operating table in between. This advancement provides greater flexibility in positioning the mobile magnetic field generator and the C-arm, facilitating interventions requiring highly angulated projections, which would be impossible with the movement constraints of two large, fixed devices. Furthermore, the lighter weight eliminates the need for a specialized room with reinforced flooring.

Magnetic robotic catheterization was first shown to be safe for human use in 2003 in cardiac ablation.^8^ Since then, it has been successfully applied for navigation in liver vasculature and coronary and neurovascular interventions.^6^^,^^7^^,^^9^, ^10^, ^11^ Although purely mechanical robotic navigation has previously been attempted to navigate in complex aortic endovascular procedures, this study describes navigation and stent placement in the renovisceral aorta using electromagnetic technology.^12^^,^^13^

## Methods

### Electromagnetically steerable guidewire prototype

**Fig 1:**
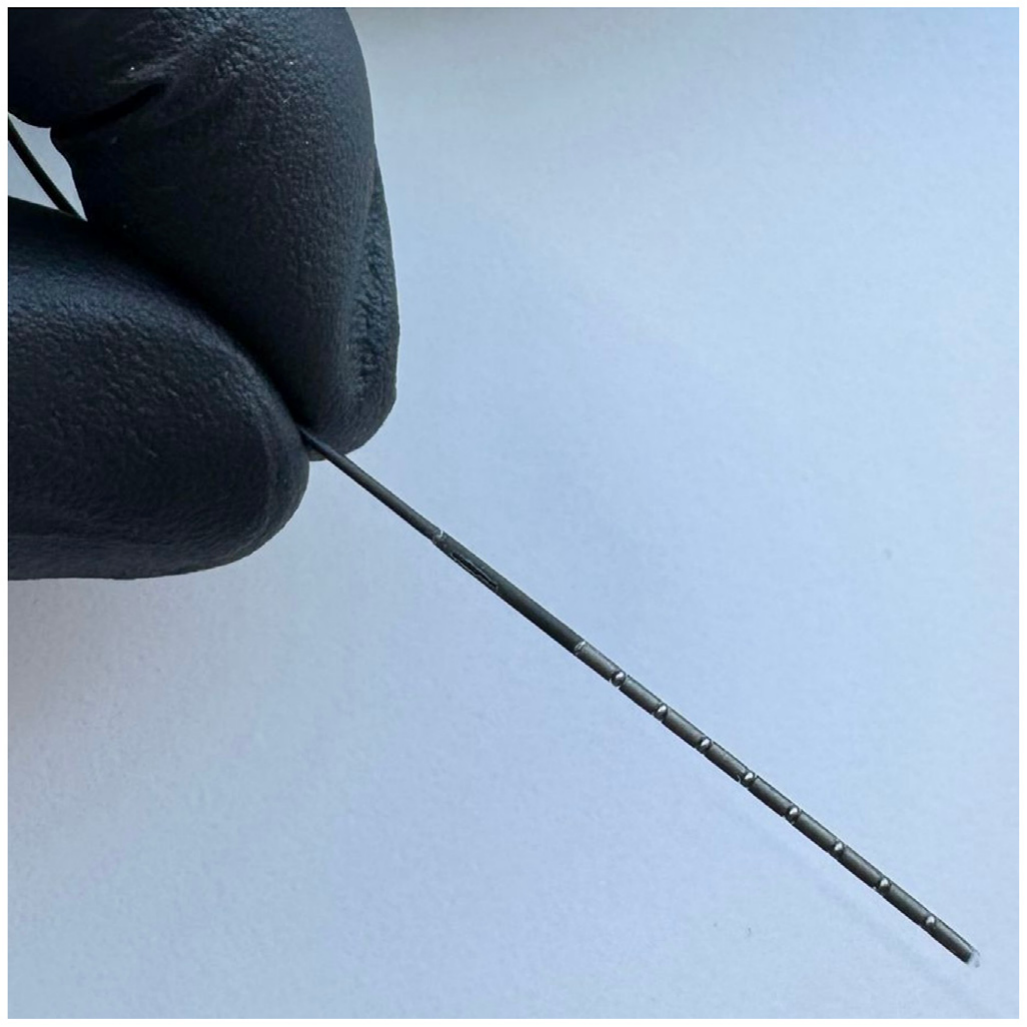
Modified 0.035” guidewire with a 23.5-mm steerable magnetic tip, consisting of neodymium-iron-boron (NdFeB) magnets and steel spheres, encapsulated in Pebax 35D.

The tip of a commercially available 0.035” stiff guide wire (GLIDEWIRE Stiff GS3503, Terumo) was modified with small magnets to make it steerable in an electromagnetic field. The magnetic tip is 23.5 mm long and comprises nine cylindrical neodymium-iron-boron (NdFeB) magnets with an outer diameter of 0.75 mm and a length of 2 mm each, spaced by steel (52,100 alloy) spheres with an outer diameter of 0.66 mm. The first millimeter of the distal end of the guide wire was cut off, the most proximal magnet glued to the cut surface, and the assembly was thereafter permanently incapsulated in a thermoplastic elastomer jacket (Pebax 35D) (Fig 1). The diameter of the modified tip was the same as the commercially available guide wire, ensuring compatibility with 0.035” catheters. The 0.035” platform was chosen due to its widespread clinical use and because the larger diameter facilitates integration of the magnetic components and thermoplastic encapsulation. For initial testing and prototyping, the above-mentioned guidewire was selected based on a favorable balance between flexibility and stability. However, the underlying principle is theoretically applicable to other guidewire platforms. Magnetically steerable guidewires with integrated magnetic elements are currently under development, aiming to enable standardized manufacturing, improved mechanical integration, and seamless compatibility with the electromagnetic navigation system (eMNS) system for future clinical application.

### Electromagnetic navigation system

**Fig 2:**
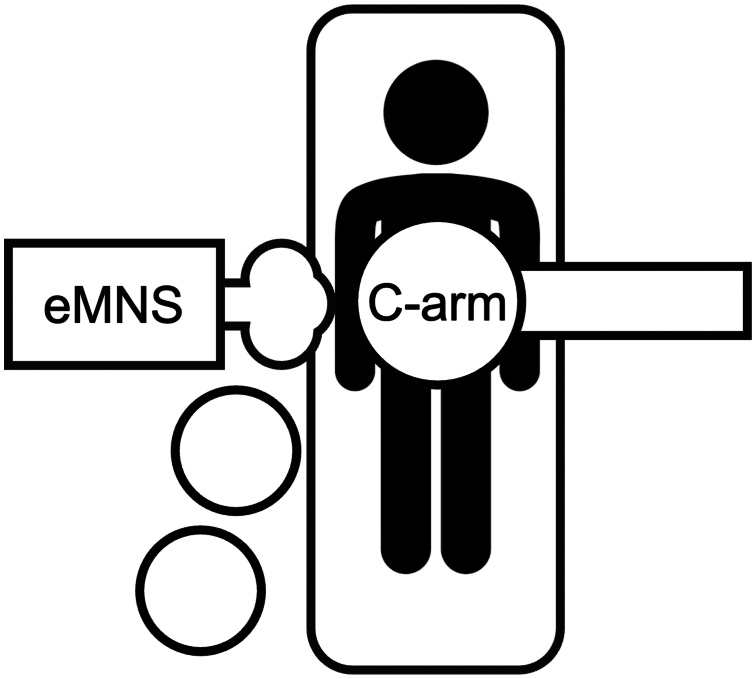
Setup for abdominal procedures with the electromagnetic navigation system (*eMNS*) and operators positioned right of the patient for femoral access; the C-arm is placed contralaterally for imaging.

**Fig 3:**
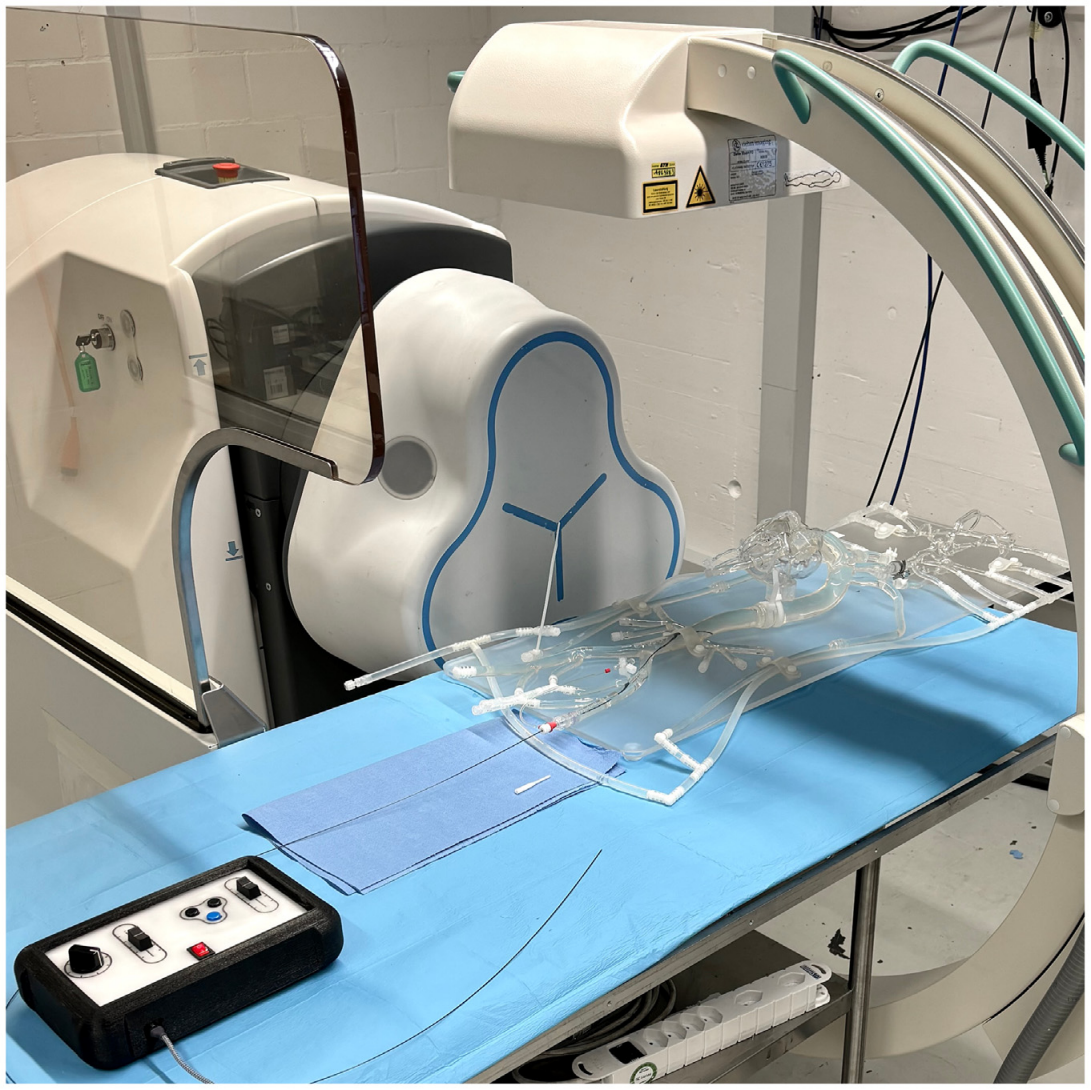
Experimental setup of magnetic field generator, steering console, aortic silicone model, and fluoroscopy C-arm.

The preclinical eMNS used in this study has previously been described.^10^ The eMNS was developed by the ETH Zurich (Multi-Scale Robotics Lab ETH Zurich) and is currently being commercialized by Nanoflex Robotics (Nanoflex Robotics AG). The system comprises three electromagnets that can each carry up to ± 35 A of electrical current. Depending on how much current is run through each of the coils, the direction and amplitude of the magnetic field can be precisely controlled in a workspace of 200 × 200 × 400 mm in front of the eMNS. In this workspace, the field can be freely controlled with at least 5 mT in magnitude and can have a magnitude as high as 25 mT at 200 mm distance from the electromagnets. Furthermore, the magnetic workspace can be moved by placing the mobile system in a desired location as well as by adjusting the height of the three electromagnetic coils using its motorized mechanism for vertical adjustment. Although the eMNS is designed for easy repositioning, it remained stationary throughout the entire experimental procedure. The operators were positioned to the right of the eMNS (Fig 2). The target area, consisting of the juxtarenal aorta and its visceral branches, was fully contained within the magnetic workspace. The working distances from the surface of the electromagnets ranged from approximately 18 to 26 cm, with the center of the juxtarenal aorta located about 22 cm from the generator. This setup resulted in adequate magnetic field strength for reliable and precise guidewire control throughout the procedure. To control the direction of the magnetic field and thus the orientation of the magnetic guide wire (MGW), the operators were provided with a console (Fig 3). A computer then processed the inputs from the console, using them to calculate the appropriate currents needed in the coils of the eMNS to achieve the desired field orientation.

### Experiment in a silicone model

**Fig 4:**
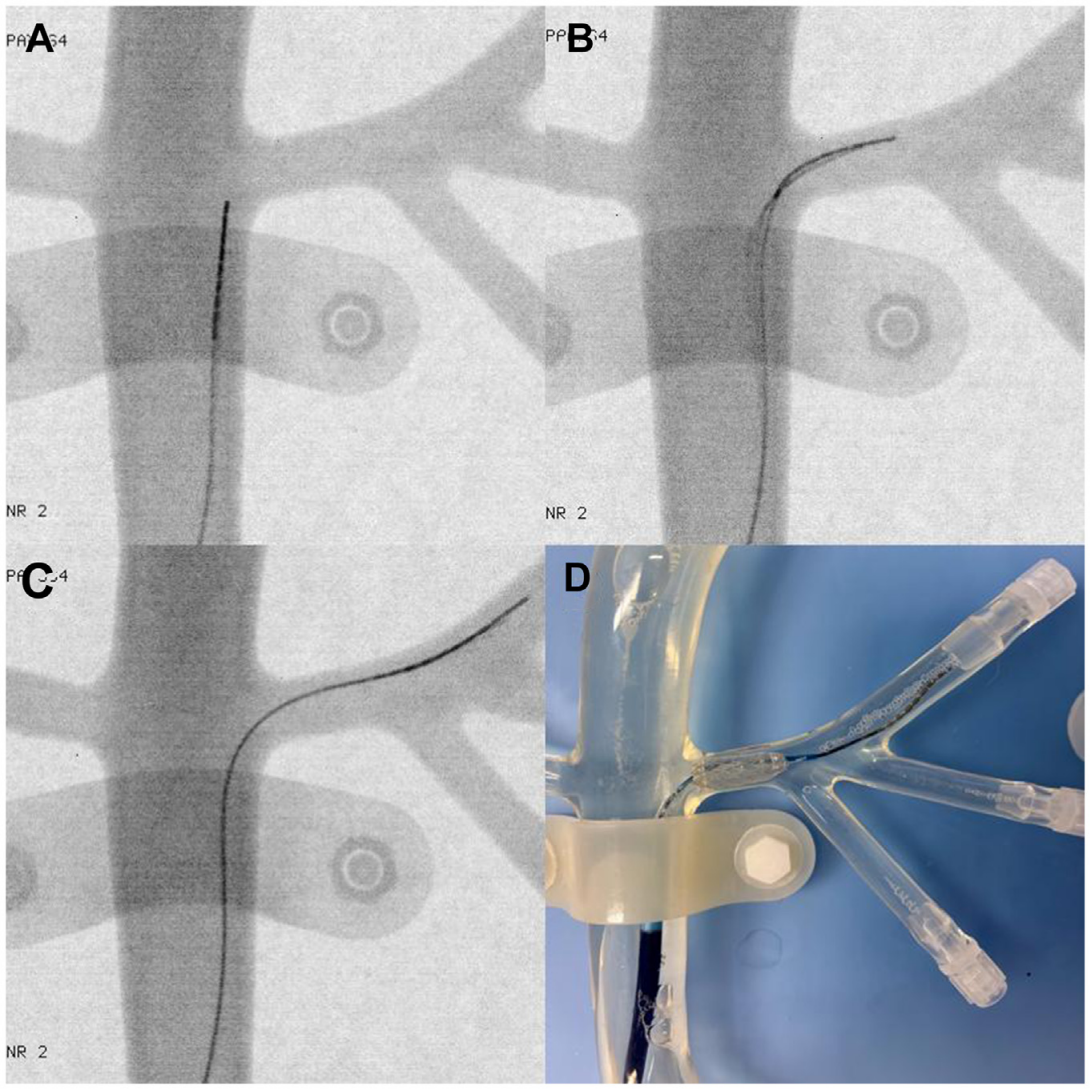
Three-dimensional printed silicone aortic model with magnetic guidewire being advanced into left renal artery **(A**, **B**, and **C)** for the purpose of deploying a balloon-expandable covered stent **(D)**.

In a three-dimensional silicone model of the human aorto-iliac arteries (TrandoMed 3D Inc), access to the right external iliac artery was obtained via retrograde introduction of a sheath (Flexor Ansel Guiding Sheath 6 F 45 cm straight tip G44154, Cook Medical) through the common femoral artery. Under fluoroscopic control (Vision FD Mobile C-Arm, Ziehm Imaging), a 0.035” MGW was manually advanced to the juxtarenal aorta. The tip of the modified guide wire was then remotely bent towards the left renal artery by adjustment of the electromagnetic field using a remote controller (Fig 4, *A*). This way, the guidewire was advanced into the artery with manual axial push until a stable position was achieved (Fig 4, *B* and *C*). This step was performed using only the guidewire itself without any auxiliary devices such as support catheters. Then, using the guidewire for support and the dilator, the sheath was subsequently advanced to a few centimeters into the renal artery using only the stability of the wire itself and the electromagnetic field to hold it in place. After removal of the dilator, a balloon-expandable covered stent (BeGraft 6x28 mm BGP2806_1, Bentley Innomed) was positioned for deployment in the ostium of the right renal artery (Fig 4, *D*). A timer was started upon insertion of the wire into the prepositioned sheath and stopped after achievement of the final position for stent deployment.

For comparison, the aforementioned steps were repeated utilizing conventional means to catheterize the renal artery. With an unmodified 0.035” guidewire (GLIDEWIRE Stiff GS3503, Terumo) for support, the 6 F guiding sheath was placed slightly distally of the left renal artery. After removal of the dilator from the sheath, a 4 F angled guide catheter (Berenstein 46,535BER, Merit Medical) was introduced over the guidewire to steer it into the right renal artery until a stable position was reached. The guide catheter was then removed again, and the sheath was positioned in the renal artery using the guidewire and dilator for support. Positioning of the balloon expandable stent for deployment and timing were performed in an identical manner for both groups.

At the end of every procedure, a fluoroscopic control was performed showing correct stent placement and patency. Finally, all devices were safely removed from the aortic model. We performed an alternating series of 20 procedures employing either a conventional guidewire (CGW) and guide catheter or the MGW and no guide catheter. The procedures were performed by an experienced endovascular surgeon and a vascular surgery trainee under the supervision of robotics engineers. A training period of 5 minutes was allocated to familiarize the operators with the magnetic navigation system prior to the start. The primary endpoint included the procedure time, which was recorded from the initiation of vascular access (insertion of the guide wire into the pre-positioned sheath) to successful positioning of the balloon-expandable covered stent. Additionally, secondary endpoints consisted of the individual registration of fluoroscopy time and dose for each experimental run.

### Statistical analysis

Statistical analyses were conducted using GraphPad Prism 10.2.2 (GraphPad Software). Descriptive statistics, including means and standard deviations, were calculated. To investigate the impact of the independent variable (use of MGW) on the pre-specified endpoints, Mann-Whitney *U* tests were conducted. Given the non-normal distribution of values resulting from a learning curve, the Mann-Whitney *U* test was deemed appropriate. No violations of the assumptions were identified. The significance level was set at α = .05, with two-tailed tests employed.

## Results

A total of 20 procedures were performed, of which 10 were with the use of a MGW for renal artery catheterization and 10 with a CGW. Technical success, defined as correct placement of the covered stent, was 100% for all procedures. The eMNS operated without technical complications, and the electromagnetic navigation of the MGW into the renal artery was effective in all experimental runs.

**Fig 5:**
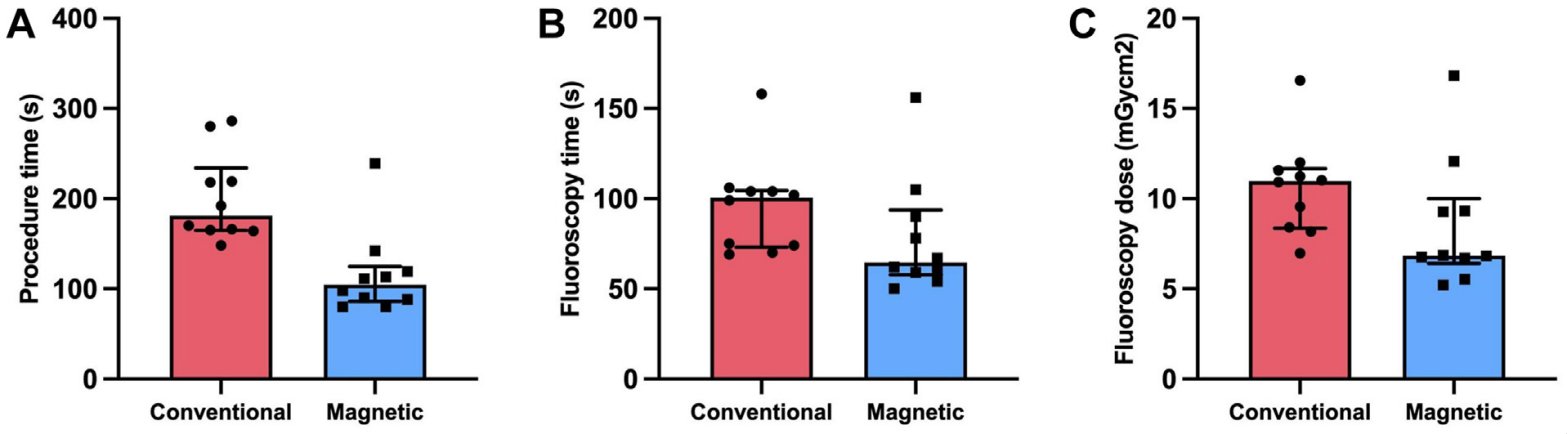
Median procedure time in seconds **(A)**, fluoroscopy time in seconds **(B)**, and fluoroscopy dose in mGycm^2^**(C)** with interquartile range for conventional and magnetic guide wire.

**Fig 6:**
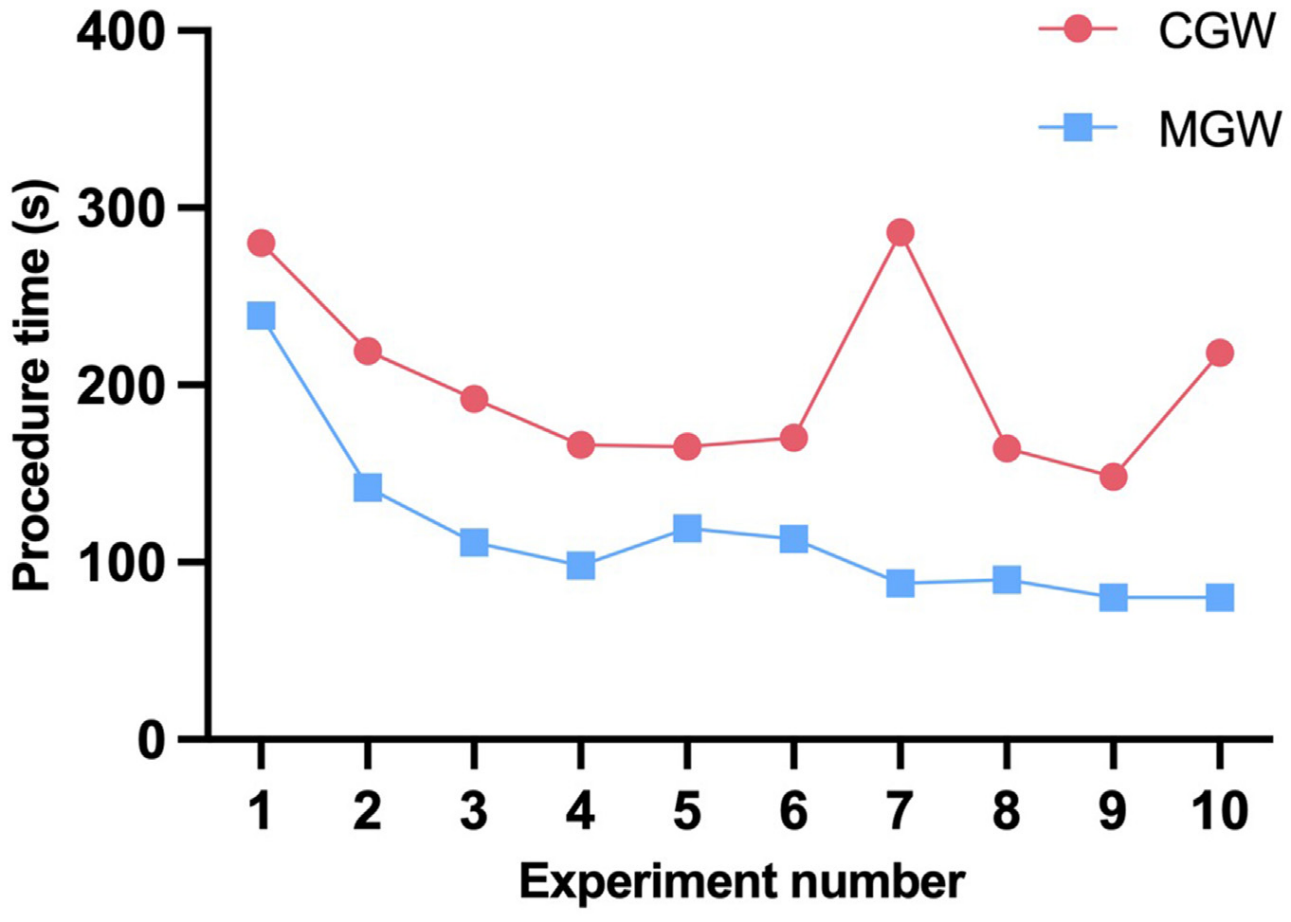
Learning curve in procedure time in seconds for each consecutively repeated experiment with conventional (*CGW*) and magnetic guide wire (*MGW*).

**Fig 7:**
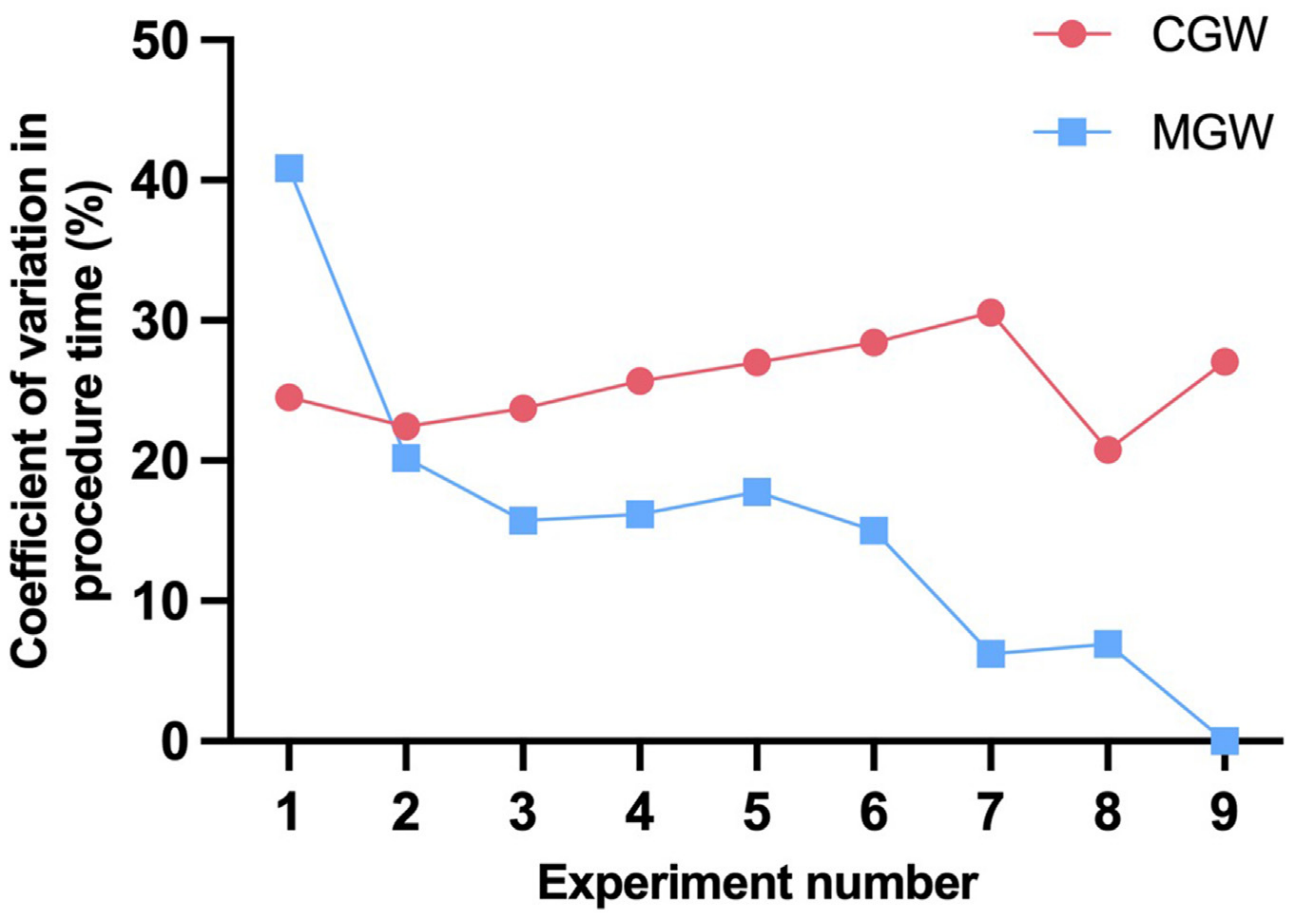
Coefficient of variation in % over time for procedure time of each consecutively repeated experiment with conventional (*CGW*) and magnetic guide wire (*MGW*) (censored for last experiment for statistical reasons).

Median procedure time was significantly shorter for MGW than for CGW (105 vs 181 seconds; *P* < .001). Furthermore, a trend for a lower median fluoroscopic time (65 vs 101 seconds; *P* = .06) and for a lower median fluoroscopic dose for MGW than for CGW (6.84 vs 10.98 mGycm^2^; *P* = .063) could be observed (Fig 5). A learning curve could be observed for both techniques, with the MGW demonstrating a steeper learning curve when compared with the conventional technique (Fig 6). Variance in procedure time decreased over time for the MGW, whereas it remained consistently high for the CGW (Fig 7).

## Discussion

Our study investigated the use of a MGW with a novel mobile electromagnetic field generator for endovascular interventions, comparing its performance with conventional means. The main advantage of the system under investigation is the flexibility of the magnetic field generator. prototyping, the above-mentioned guidewire was selected based on a favorable balance between flexibility and stability. However, the underlying principle is theoretically applicable to particularly those who employ permanent magnets, which often require multiple heavy units; the current device is a single-unit system with only one electromagnetic field generator, which can be easily repositioned. This design significantly simplifies clinical implementation in complex endovascular aortic surgeries.

Pioneering mechanical devices such as Sensei and Magellan (Hansen Medical Inc) were used for cardiac ablative procedures and even attempted for selected steps of complex aortic procedures such as branched endovascular aortic repair (EVAR) and fenestrated EVAR.^12^^,^^13^ However, they were eventually discontinued due to issues with cost and integration.^14^ Their successors, CorPath GRX (Siemens Healthineers) and R-One (Robocath), offered advantages like compatibility with standard catheters and wires, remote steerability, and greater versatility in applications.^15^ Despite these improvements, being purely mechanical, they still fall short in terms of precision, handling complex anatomies due to limited catheter motion, and vessel injury when compared with more recent devices using magnetic navigation technology^14^^,^^16^^,^^17^

With magnetic systems like Niobe ES or the newer Genesis RMN (Stereotaxis Inc) it is possible to remotely steer the magnetic tip of a specially prepared guidewire inside a vessel. They offer advanced capabilities for precise navigation when compared with purely mechanical navigation, but have significant space requirements, limiting C-arm and patient positioning.^11^^,^^17^ Additionally, their heavy weight requires a specialized room with reinforced floors, and the strong permanent magnetic fields require magnetic shielding to mitigate risks to nearby medical devices and patients.^18^^,^^19^

In contrast, the eMNS used in our study is lighter and mobile. It can be positioned adjacent to the supine patient and placed on either side, depending on room layout and operator access. Although the current field generator may still impose some limitations on C-arm mobility, this is less critical in cerebrovascular procedures, where the system can be positioned at the head of the patient. Future iterations are expected to further improve flexibility and ergonomics (for example, by decoupling the field generator from the base unit and connecting them via a cable for power and coolant circulation). Ultimately, integration of the coils into existing imaging systems such as a C-arm is envisioned. Based on current field strength performance, this appears technically feasible and would greatly enhance the system’s potential for clinical integration in complex aortic interventions. This would further enhance the chances of being integrated into clinical practice in complex endovascular aortic surgery.

The results revealed possible significant advantages. Notably, three-dimensional control of the guidewire was readily achievable under two-dimensional fluoroscopic guidance without the need for additional steps or visualization aids. Proficiency in the use of the MGW was quickly attainable, showing an even steeper learning curve than with the CGW, despite the operator’s prior experience in conventional endovascular techniques. Interestingly, only a short familiarization period of 5 minutes was required before operators were able to use the system effectively. Neither operator had prior experience with the magnetic navigation system, and its intuitive control interface likely contributed to the rapid adaptation. However, the study design included only two individuals and lacked inter-observer variability, which may limit the generalizability of these findings. Moreover, althoough basic tasks such as renal artery cannulation could be performed after minimal training, more complex procedures will likely require significantly more experience to ensure safe and effective use. To address this, a follow-up study is planned to systematically assess the learning curve across varying levels of clinical experience, including medical students, junior endovascular physicians, and senior specialists.

The aim of our study was to investigate potential advantages of a MGW in difficult arterial anatomy because this can significantly impact catheterization success, often resulting in time-consuming and radiation dose-intensive procedures. In conditions such as ischemic stroke, where reported catheterization failure rates of up to 5.1% underscore the severity of these challenges, the impact on clinical outcomes can be substantial.^2^ Similarly, in complex endovascular repair, such as those involving reno-visceral arteries, catheterization can be exceedingly challenging. Catheterization with standard equipment has reported failure rates of up to 53%, necessitating the frequent use of more advanced devices like steerable sheaths.^3^^,^^20^ The steep arterial angles encountered in a femoral approach may even necessitate upper extremity arterial access, which carries the risk of perioperative stroke.^3^ To simulate these conditions, a three-dimensional silicone aortic model was selected. Previous research has demonstrated a correlation of a surgeon’s experience in endovascular procedures and performance in a model of this kind, making it a suitable surrogate for real-life procedures.^21^ The results of our study suggest that employing a MGW under these conditions can deliver success rates comparable to the CGW, thereby reducing the need for cost-intensive auxiliary devices or access sites prone to complications.

Particularly notable in our study was the significantly shorter procedure time with less variation over time when employing the MGW. This can most likely be attributed to the fact that repositioning the MGW required significantly fewer steps, as it combines multiple features such as steerability and support into a single device. A shorter procedure time in endovascular repair has been associated with a lower complication rate and perioperative mortality.^22^ Furthermore, it can lead to a significant reduction in treatment cost, especially considering that procedures performed in a hybrid operating room are inherently cost-intensive, with a conservatively estimated cost per minute of EUR 19.88—more than double that of a conventional operating room (EUR 9.45).^23^ In standard EVAR, operating time alone can result in costs ranging from EUR 1813 to 2797.^24^^,^^25^ These costs are most likely even higher for more complex endovascular aortic repairs, where procedural durations can extend up to 6 to 8 hours.^26^^,^^27^ Additionally, shorter procedure times could facilitate scheduling more cases within the same day, potentially compensating capacity constraints arising from limited operating personnel or other economic restraints. The utilization of a MGW also reduces the need for auxiliary devices such as differently shaped wires, guide catheters, and steerable sheaths. This does not only simplify the procedure but also holds the potential for substantial savings in supply cost. For instance, in a 2019 study examining non-ruptured EVAR treatment in the United States (U.S.), supply cost only amounted to as much as U.S. $19,275.^28^ Similarly, in a 2016 study on endovascular treatment of chronic occlusive peripheral arterial disease, supply costs could constitute up to 40% of the total procedure costs, which were estimated at U.S. $7800.^29^ In conclusion, the adoption of MGW technology potentially presents an opportunity for a reduction in procedure time and demand for expensive devices. This, in turn, could aid in gradually offsetting the initial investment for the field generator and ultimately contribute to overall cost-effectiveness in endovascular procedures. Although the initial investment in the eMNS system may be substantial, it is expected to be in the range of a high-end monoplanar C-arm system, such as the Cios Alpha (Siemens Healthineers), and thus within a familiar cost category for many institutions. The magnetically steerable guidewire is also anticipated to be comparable in price to premium non-steerable guidewires currently used in clinical practice, such as the Aristotle Colossus Guide Wire (Scientia Vascular).

Lastly, our findings indicated a substantial trend for a potentially lower fluoroscopy time and dose for employment of the MGW when compared with the conventional method. This can be explained by the lack of time-consuming exchanges of different guide catheters and wires, which often need to be performed under fluoroscopic guidance. However, due to the limited number of repeated measures (10 per experimental arm), the study was underpowered to yield significant results for these secondary endpoints.

Future research plans should therefore include larger experiments with more operators to reduce inter-observer effects, thereby enhancing the generalizability and robustness of the findings. Furthermore, we should aim to validate these findings under more lifelike conditions, such as animal models, to better replicate the complexities of human anatomy and physiology. This will help determine the full potential of the MGW with the mobile electromagnetic field generator for endovascular applications and assess its effectiveness and safety in a clinical setting.

## Conclusions

Precise control of a steerable guidewire with a mobile electromagnetic field is technically feasible in a realistic silicone-model of the human aorta. In this study it facilitated *in vitro* renal artery catheterization and stenting faster than with conventional means and with fewer auxiliary devices. This novel technology presents numerous potential therapeutic applications in the future of endovascular interventions, offering the prospect of reduced procedure time, better patient outcome, and potentially lower costs. However, additional testing for various endovascular procedures under more life-like conditions is necessary.

## Author contributions

Conception and design: CZ, JL, SV, VM, DK, SJ

Analysis and interpretation: CA, JL, SV, SJ

Data collection: CA, JL, SV, SJ

Writing the article: CZ, SJ

Critical revision of the article: JL, SV, VM, DK, SJ

Final approval of the article: CZ, JL, SV, VM, DK, SJ

Statistical analysis: CZ

Obtained funding: DK, SJ

Overall responsibility: SJ

DK and SJ contributed equally to this article and share co-senior authorship.

## Funding

This work was funded through departmental resources.

## Disclosures

J.L. and S.V. were involved in the development of the technology and are now employed by Nanoflex Robotics AG, Switzerland, which aims to commercialize it and provided the investigational device.
